# Three-Dimensional Structure and Disposition of the Air Conducting and Gas Exchange Conduits of the Avian Lung: The Domestic Duck (*Cairina moschata*)

**DOI:** 10.1155/2014/621982

**Published:** 2014-02-05

**Authors:** A. N. Makanya, B. M. Kavoi, V. Djonov

**Affiliations:** ^1^Department of Veterinary Anatomy & Physiology, University of Nairobi, Riverside Drive, P.O. Box 30197, Nairobi 00100, Kenya; ^2^Institute of Anatomy, University of Bern, Baltzerstrasse 2, 3000 Bern, Switzerland

## Abstract

The anatomy of the domestic duck lung was studied macroscopically, by casting and by light, transmission, and scanning electron microscopy. The lung had four categories of secondary bronchi (SB), namely, the medioventral (MV, 4-5), laterodorsal (LD, 6–10), lateroventral (LV, 2–4), and posterior secondary bronchi (PO, 36–44). The neopulmonic parabronchi formed an intricate feltwork on the ventral third of the lung and inosculated those from the other SB. The lung parenchyma was organized into cylindrical parabronchi separated by thin septa containing blood vessels. Atria were shallow and well-fortified by epithelial ridges reinforced by smooth muscle bundles and gave rise to 2–6 elongate infundibulae. Air capillaries arose either directly from the atria or from infundibulae and were tubular or globular in shape with thin interconnecting branches. The newly described spatial disposition of the conducting air conduits closely resembles that of the chicken. This remarkable similarity between the categories, numbers, and 3D arrangement of the SB in the duck and chicken points to a convergence in function-oriented design. To illuminate airflow dynamics in the avian lung, precise directions of airflow in the various categories of SB and parabronchi need to be characterized.

## 1. Introduction 

It has long been known that airflow in the bird lung is mainly unidirectional [1, 2], and this has been attributed largely to the bellows-like action of the air sacs. In a recent report, it has been demonstrated that airflow in the alligator lung is unidirectional just like in birds, despite the absence of air sacs [3]. This has thrown more confusion into the already controversial descriptions of the avian lung structure and function. Over the years, the structure and function of the avian lung have intrigued scientists and the actual structural complexity is only beginning to come to light [4].

The seminal insights into the avian lung function such as the description of unidirectional air flow [2, 5] and cross-current gas exchange [6] were established in the duck lung. While several studies have attempted to elucidate the fine details of the avian lung structure, certain aspects that could be directly related to function still remain enigmatic and several techniques including 3D reconstruction have been attempted to resolve the spatial arrangement of the gas exchange tissue [7–9]. Furthermore, lung structure among vertebrates has been most refined in birds where the thinnest blood gas-barrier is encountered [10, 11].

Generally, the avian lung is reported to be noncompliant and ventilation is accomplished by the bellows-like action of the air sacs [12, 13]. In a recent study, the definitive structure of the lung of the domestic fowl has been documented with the notion that four categories of secondary bronchi are present [4]. It has previously been noted that literature on the anatomy of the avian lung is vast, confusing, and contradictory [14], but the picture in the extensively studied domestic fowl has recently been elucidated [4, 15, 16].

In the current study, the duck lung has been chosen since it has been previously used largely in the study of the lung function [5, 6, 17–19] and also in 3D elucidation of the gas exchange structure (Woodward and Maina, 2008). Much controversy has been mainly on the nomenclature, numbers, and distribution of the secondary bronchi and how their arrangement affects gas flow in the lung [4]. The recent finding that gas flow in the alligator is unidirectional in total absence of air sacs [3] has heightened the need to probe the avian lung further and also underpins the fact that the 3D arrangement of the major air conduits (secondary bronchi and parabronchi) is paramount. We have previously shown the categories of the venous and arterial vessels at the parabronchial level and have demonstrated that the exchange blood capillaries are largely orthogonal to the air capillaries, thus establishing a cross-current system at the gas exchange interface [16]. The difficulties in dual visualization of both the air and blood capillaries, even with 3D reconstruction, have previously been demonstrated (Woodward and Maina, 2008).

In previous studies of the avian lung structure, subtle differences have been noted in the general arrangement of the air conduits among species [20], but a clear comparative database is elusive mainly due to the disparities in the anatomical descriptions of the various structures. The descriptions of the secondary bronchi in literature, for example, have been most confusing in nomenclature, the structure, spatial distribution, and numbers. In the domestic fowl, different names and numbers of the various secondary bronchi have previously been proposed by contemporary investigators [20–22]. The situation, however, has recently been clarified with the notion that there are 4 MV, 7–10 LD, 1–3 LV and an average of 40 PO, a newly described category [4]. In the current study we have resolved this controversy with the finding that global distribution of the airways is generally similar to that of the chicken, with variations in wall structure and origin of some categories.

## 2. Material and Methods

Adult domestic ducks (*Cairina moschata*) were purchased from farmers and transported to the Department of Veterinary Anatomy and Physiology of the University of Nairobi. The animals were subjected to the selected investigative procedures as detailed below (outlined in Table 1). In all cases, anesthesia was achieved by intra-abdominal injection of Euthatal (sodium pentobarbitone, injected i.p. at a dosage of 50 mg kg^−1^ body mass). All protocols were approved by the Animal Care and Use Committee of the University.

### 2.1. Tissue Fixation

Lungs intended for microscopy were fixed in situ by intratracheal infusion with a solution of 2.5% glutaraldehyde in 0.1 M cacodylate buffer (pH 7.4, 350 mOsm) and subsequent immersion into the same fixative.

### 2.2. Macroscopic Observations

Lungs were carefully dissected out by cutting off the ribs. After a general observation, a longitudinal cut was made on the ventral aspect of the lung along the long axis of the intrapulmonary primary bronchus, thus revealing the internal aspect of the mesobronchus. Identification and enumeration of the various categories of secondary bronchi emerging from the primary bronchus was conducted. In addition, lungs were subjected to macrophotography using a digital camera.

### 2.3. Light and Transmission Electron Microscopy

Fixed lungs were sliced into slabs and then diced into small blocks. Tissue blocks were postfixed in osmium tetroxide; block-stained with uranyl acetate, dehydrated through ascending concentrations of ethanol, and embedded in epoxy resin. Semithin sections were obtained at a nominal thickness of 1 *μ*m, stained with toluidine blue, and viewed under a Leica DMBR digital light microscope. Ultrathin sections were obtained at 90 nm, counterstained with lead citrate, and viewed on a Philips CM-12 transmission electron microscope.

### 2.4. Intratracheal Casting

#### 2.4.1. Methylmethacrylate Resin Casting

Methylmethacrylate resin was introduced through the trachea until the whole airway system was filled with the resin. At least 1 h after perfusion, the lungs were dissected out and digested in 15% KOH for 3-4 weeks. The specimens were then processed further for the scanning electron microscope as detailed below.

#### 2.4.2. Silicon Rubber Casting

Silicone rubber was mixed with silicone oil at the ratio of 5 : 2 (volume per volume, v/v). The resulting mixture was mixed with hardener at the ratio of 1 : 35 (hardener to mixture, v/v). Blue dye was then added and stirred until the required color was obtained. The mixture was then injected into the tracheae of adult ducks under deep barbiturate anesthesia (Euthatal). The silicone was left to set for at least 15 min and the lungs were dissected out and corroded in 15% potassium hydroxide. Photographs of the required parts of the resulting silicone casts were obtained with a digital camera.

### 2.5. Scanning Electron Microscopy

The lungs were fixed by intratracheal infusion of glutaraldehyde fixative as outline above. Selected lung specimens were dehydrated through ascending concentrations of ethanol, critical point-dried in liquid carbon dioxide, and mounted on aluminum stubs. The specimens were sputter-coated with gold and viewed under a Philips XL 30 FEG scanning electron microscope.

## 3. Results

**Figure 1:**
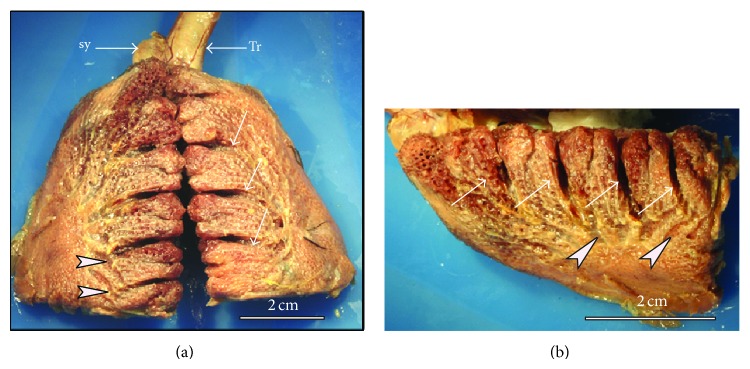
Macrographs of fixed lung specimens showing the dorsolateral aspect of the duck lung. ((a) and (b)) On the dorsolateral surface the rib impressions (arrows) extend down to the level where the laterodorsal secondary bronchi (LD) become visible below the parietal pleura (arrowheads). The trachea (Tr) and the syrinx (Sy) are also shown. (b) Close-up of the lateral view of the right lung showing the superficial disposition of the LD (arrowheads). Arrows indicate costal sulci.

**Figure 2:**
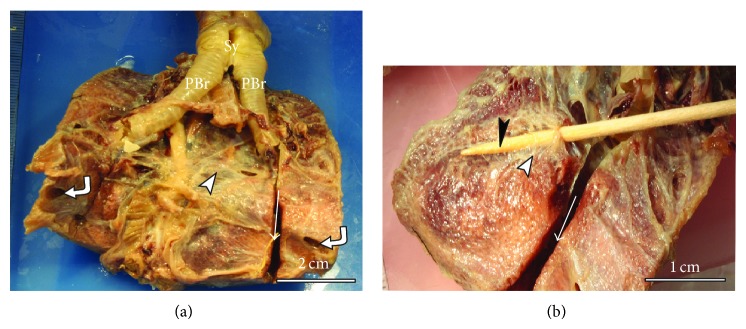
Macrographs of fixed lung specimens showing the medioventral aspect of duck the lung. (a) On the ventral surface the ventral septum covers the lung (arrowhead) and the ostia of posterior thoracic air sacs (curved arrows in (a)) are shown. The primary bronchi (PBr) and the syrinx (Sy) are also indicated. (b) Ventral view of the lung with a splint of wood (black arrowhead) inserted into the fourth medioventral secondary bronchus (MV4) to show the superficial position of this secondary bronchus (white arrowhead). The mesobronchus has been opened (arrow) to show its trajectory.

**Figure 3:**
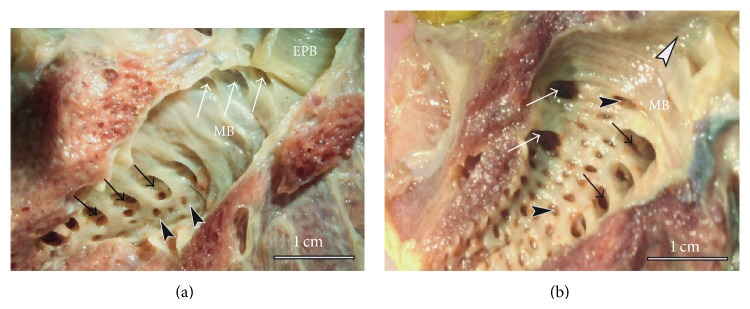
Macrographs of fixed left lung specimens with the mesobronchus opened to show the various categories of secondary bronchi. (a) The white arrows show the openings of MV at the anterior part of the mesobronchus (MB) and just behind the extrapulmonary primary bronchus (EPB). Black arrows indicate laterodorsal secondary bronchi (LD) and the arrowheads point to PO. (b) A close-up of the internal aspect of the mesobronchus showing the openings to the lateroventral (LV) bronchi (white arrows), on the lateral aspect and opposite those of the LD (black arrows). The dark arrowheads denote the openings to the very numerous posterior secondary bronchi (POs) while the white arrowhead points to the cranial direction.

The gross appearance of the adult duck lung is demonstrated in Figures 1–3. The lung has a trapezoid shape with a sharp ventral border. The dorsal border is interrupted by deep costal sulci while the medial surface was generally smooth and flat. On the lateral aspect, the terminal parts of the laterodorsal secondary bronchi (LD) were superficially positioned just below the parietal pleura and were as such discernible (Figure 1). Similarly, the lateroventral secondary bronchi (LV) emanating from the fourth medioventral bronchus (MV4) were visible on the ventral aspect of the lung (Figure 2).

Identification and enumeration of the various categories of the secondary bronchi were done on lung specimens, in which the mesobronchi had been opened to reveal the openings of such bronchi (Figure 2) as well as in silicone rubber casts. Incidentally, the LV emanating from MV4 were more superficial and were covered by a thin transparent membrane and as such could be counted from the ventral surface of the lung or by opening the MV4 longitudinally.

**Figure 4:**
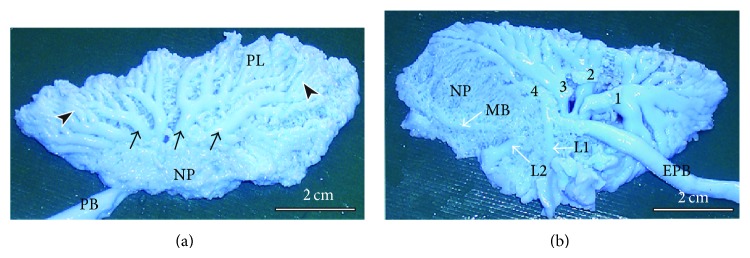
Silicon rubber casts showing the disposition of the secondary bronchi and parabronchi in the duck lung. (a) Dorsolateral view showing the LD (dark arrows) emerging at the junction between the neopulmonic (NP) and paleopulmonic (PL) regions. The LD gain a superficial position, curve dorsally and course towards the dorsal border giving rise to parabronchial branches that meet and anastomose with those from the MV. Note that the neopulmonic region (NP) takes the ventral third of the lung and extends the entire length of the lung. Dark arrowheads denote the branches of LD and EPB denotes the extrapulmonary primary bronchus. (b) On the ventral aspect, the 4 MV (1–4) are clearly visible, the first one coursing cranially, the second dorsally, and the last two caudally. Notice also a lateroventral secondary bronchus (L1) emerging from the MV4 while the second one (L2) emerges from the mesobronchus (MB). The MV1 serves the craniomedial aspect of the lung, MV2 and MV3 the dorsomedial and caudal medial aspects, respectively, while MV4 largely supplies the neopulmonic (NP) region.

Details on the mean numbers and ranges of the various categories of SB are provided in Table 2. The PO were most numerous (36–44, 39 ± 2) followed by LD (7–10, 9 ± 0.9); the MV (4-5, 4.3 ± 0.5) and the LV (2–4, 3.2 ± 0.9) were the least in number (data provided as ranges and as means ± SD, resp.). About half the LV were mesobronchial (emanated from the mesobronchus) while the rest were bronchial (emanating from MV4, see Figure 3). The numbers of the secondary bronchi closely approximated those of the chicken lung (Table 3). The 3D disposition of the parabronchi and secondary bronchi was better visualized in silicone rubber casts (Figure 4) where it was noted that the neopulmo extended along the entire ventral half of the lung and that half of the first five LD were directed rostrodorsally while the rest were directed dorsocaudally. Parabronchi of the neopulmonic region formed a dense network of anastomosing tubes and inosculated those from MV4 and LV and also those from LD laterally (Figure 4) and plausibly also the ventral group of the PO.

**Figure 5:**
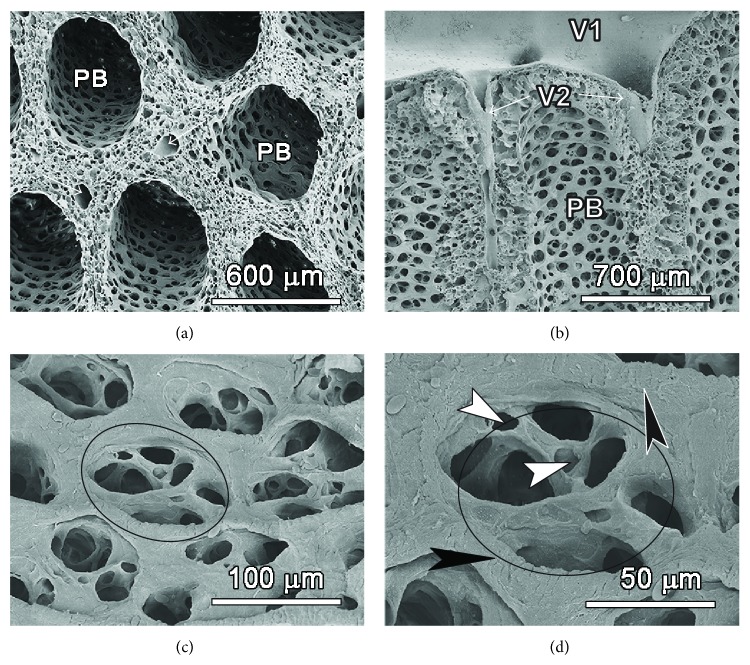
Scanning electron micrographs showing the three dimensional architecture of the smaller air conduits and gas exchanging units. (a) and (b):The parabronchi (PB) have the typical honeycomb arrangement. Inerparabronchial vessels are visible between adjacent parabronchi (arrows in (a)). The large bronchial vein (V1) receives interparabronchial veins (V2) that run in the interparabronchial space (see (b)). (c) and (d):A close-up of the internal aspect of the parabronchi shows that the atria (ellipse in (c)) are extremely shallow and have 2-6 infundibulae (IF). The atria are surrounded by a ridge of epithelial tissue (white arrows in (d)), which is reinforced by smooth muscle characteristic of interatrial septa (see Figure 7). Some air capillaries emerge directly from the floor of the atrium (white arrowheads in (d)) while others are extensions from infundibulae.

An overview of the lung parenchyma with the scanning electron microscope shows the typical honeycomb arrangement of large parabronchi with large bronchial blood vessels giving rise to interparabronchial branches that supply and drain the exchange units (Figure 5).

The interparabronchial vein receives intraparabronchial veins, the only large vessels that traverse the parabronchial mantle. The interparabronchial veins run in the interparabronchial septum and bulge into the parabronchial parenchyma where they are surrounded by connective tissue (Figure 5).

**Figure 6:**
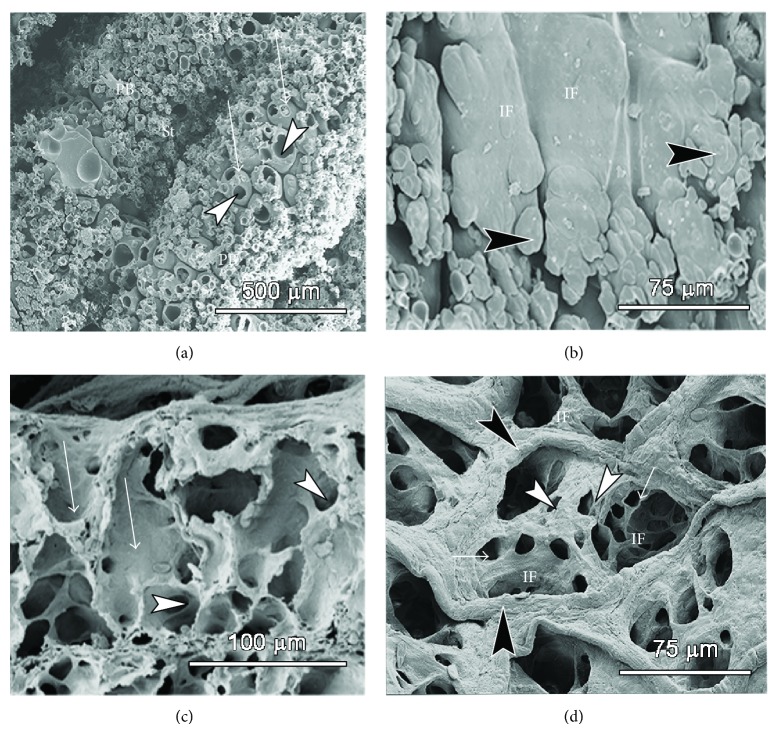
Scanning electron micrographs showing the three-dimensional architecture of the smaller air conduits and gas exchanging units. (a) and (b): Scanning electron micrographs of mercox casts (intratracheal infusion) showing the parabronchi (PB) with numerous infundibulae (arrowheads in (a), IF in (b)), some of which are raptured (arrowheads in (a)) due to presence of trapped air. Dark arrowheads in (b) denote globular air capillaries. (c) and (d): Micrographs of critical point-dried tissue showing a longitudinal view of the exchange units and a bird's eye view (d) of the same. The atria are shallow but the infundibulae are long (white arrows in (c)) and give rise to numerous air capillaries (arrowheads in (c)). The atria are surrounded by a ridge of epithelial tissue (dark arrows), which is reinforced by smooth muscle characteristic of interatrial septa (see also Figure 7). Some air capillaries emerge directly from the floor of the atrium (white arrowheads in (d)) while others (white arrows in (d)) are extensions from infundibulae (IF).

**Figure 7:**
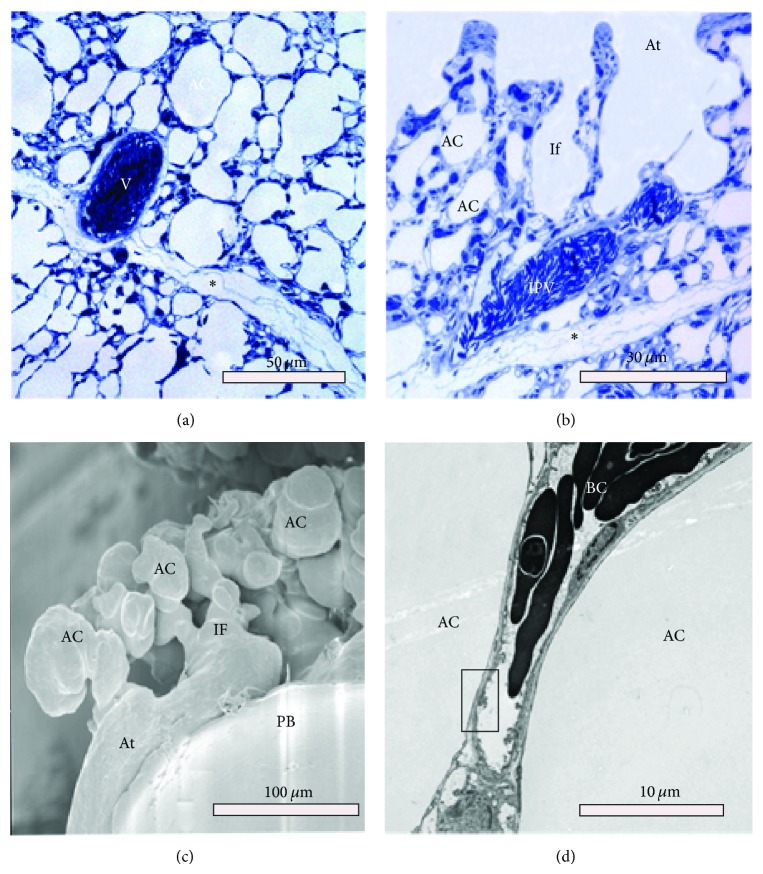
Micrographs showing the structure of the parabronchial parenchyma ((a) and (b)) and the ultrastructural details of the air capillaries (c and d). (a) Adjacent parabronchi are separated by a thin interparabronchial septum (asterisk). Large blood vessels (V) in the vicinity of the septum bulge into the parenchyma. (b) The only blood vessel that traverses the parabronchial mantle is the intraparabronchial vein (IPV) shown here. Notice (∗) outside the interparabronchial septum (∗) and air capillaries (AC) infundibulae (If) and atria (At). (c) The mantle consists of shallow atria (At) leading to elongated infundibulae (IF) and globular air capillaries (AC). (d) TEM micrograph showing an exchange capillary BC with a thin blood-gas barrier (rectangle) separating air capillaries (AC) from the lumen of the blood capillary.

On the internal aspect, the parabronchi show that the atria are extremely shallow and have 2–6 infundibulae and a few air capillaries emanate directly from the floor of the atria (Figure 5) while others are continued from the rather elongate infundibulae (Figures 6 and 7). The atria are surrounded by a ridge of epithelial tissue, which is reinforced by smooth muscle characteristic of interatrial septa (Figure 7; see also Figure 6).

Intratracheal Mercox casts (Figure 6) show the disposition of the numerous infundibulae on the parabronchi. Some of infundibulae and air capillaries appear hollow, which is due to presence of trapped air, attesting to blind ending nature of some of them. Air capillaries had a predominantly globular shape. A cross-sectional view of the exchange units and a bird's eye view (Figure 5) reveal shallow atria, elongated infundibulae that give rise to numerous air capillaries. The atria are surrounded by ridges of epithelial tissue, which are reinforced by smooth muscle characteristic of interatrial septa (Figure 6). Some air capillaries emerge directly from the floor of the atrium while others are extensions from infundibulae.

The 2D architecture of the parabronchial parenchyma is illustrated in Figure 7. The parabronchial mantle consisted of shallow atria supported by interatrial muscles and led into elongated infundibulae and globular air capillaries. The interparabronchial septum was thin and relatively uniform, measuring a paltry 1 *μ*m in some areas. The interparabronchial septa were laden with collagen fibers and completely formed both a functional and a structural boundary between conterminous parabronchi (Figure 7). Due to the small thickness of the interparabronchial septa, the large vessels bulged into the parenchyma of neighboring parabronchi. Within the parabronchial mantle blood capillaries were supported by epithelial plates formed by abutting type I cells of adjacent air capillaries (data not shown) (Figures 6 and 7).

## 4. Discussion

The difficulties in understanding the avian lung function emanate partly from poor understanding of the lung structure and partly due to the rampant distorted information in the literature [15]. In the current study, we have employed a multitechnique visualization approach to study the 3D arrangement of the air conduits. Notably, the categories of secondary bronchi and their spatial disposition closely resemble those recently described in the chicken [4].

The lung with its trapezoid shape differs slightly from that of chicken, which is rhomboid [4]. The neopulmolnic region appears to extend the entire length of the lung, forming the ventral third of the lung tissue. Other gross features of the duck lung closely resemble those of the chicken, with the notable exception of the LD and LV. The dorsal extensions of the LD were superficially placed just below the pleurae, while among the LV, the ones emanating from the mesobronchus were also visible beneath the pleura. The functional significance of this disposition of the conducting secondary bronchi is unclear, but preliminary investigations show that they are covered by thin membranes similar to those of air sacs and may be important in propulsion of air.

A quick look at Tables 2 and 3 shows that the arrangements of the secondary bronchi in the duck are not much different than those described for chicken [4]. In the chicken the MV are 4, while in the duck, they vary between 4 and 5. The disposition of the other SB is similar to that of the chicken. The first LD, however, does not supply the lower front lateral quadrant as is the case for the chicken, but this region is taken by neopulmonic parabronchi. Notably, the categories, distribution, and numbers of the secondary bronchi conform to the recently described pattern in the chicken lung [4].

Investigations on the 3D disposition of the smaller air conducting and gas exchange units in the duck lung have been previously based mainly on serial sectioning and 3D reconstruction [7, 9]. Here we have combined intratracheal casting with critical-point dried tissue to observe the spatial architecture of these smaller conduits. The atria were found to be extremely shallow, and infundibulae were large and elongate and gave rise to globular air capillaries. Interestingly, some air capillaries emerged directly from the atria. These structures had mainly globular shapes with narrow interconnections as described elsewhere [7, 9].

One of the factors thought to be responsible for the unidirectional flow of the air in the avian lung is the structure of the major bronchi [1, 23]. Hazelhoff [1] presented a hypothetical model that allowed unidirectional airflow in the bird lung based solely on the topography of the intrapulmonary bronchi (independent of the air sacs). Later on, [23, 24] insinuated that for unidirectional airflow to occur, air in the caudal part of the mesobronchus has to be directed towards the orifices of the mediodorsal (recently renamed laterodorsal) secondary bronchi [4]; this study). Proper interpretation of these earlier studies was stymied by lack of complete understanding of the categories and spatial disposition of the air conduits. Recently it has been shown that presence of air sacs is not requisite for unidirectional airflow [3], which underpins the model of Hazelhoff that emphasizes the importance of the 3D arrangement of the major bronchi [1]. Indeed it has been noted that unidirectional airflow is caused by the structural arrangement of the gas exchanging tubes themselves [25] and that this phenomenon may have evolved in preavian archosaurs, the common ancestor of crocodilians, birds and dinosaurs [3]. The remarkable similarity between the 3D arrangement of the secondary bronchi in the duck and chicken and the numbers of secondary bronchi as well (Table 3) points to a convergence in function-oriented design. While few differences such as regions supplied by some secondary bronchi and the extent of the neopulmonic region occur, the general design is the same. Other minor differences occur in the smaller conduits where in the duck, atria are generally shallow, infundibulae large and elongate, and air capillaries are globular [7, 9]. In the chicken [26] and the pigeon [17] the air capillaries appear to be more tubular and their interaction with the blood capillaries has been shown to be largely orthogonal [16, 17, 26]. The air sacs in the bird lung, while playing a crucial role in reducing the weight in favor for volancy and also blowing air into the bronchi, may not be absolutely requisite for unidirectional air flow. It has been shown that in addition to alligators, airflow in lungs of crocodiles is also unidirectional, with the implication that this is a plesiomorphic archosaurian trait [27]. Unidirectional airflow in birds is important for improved gas exchange efficiency since no mixing of gases occurs and a high diffusion gradient of exchange gases can be maintained [11]. The exact premise on which unidirectional airflow in birds has been built needs to be investigated.

## Figures and Tables

**Table 1:** Techniques applied in the study and respective numbers of animals used.

Technique	Number of animals used	Results accomplished
Macroscopic examinations	6∗	^Ψ^Enumeration of secondary bronchi
Silicone rubber casting	3	Enumeration of secondary bronchi Study of 3D arrangement of secondary bronchi
Semithin and TEM	3∗	Study of the exchange tissue and BGB
SEM on critical-point dried tissue	3∗	Study of 3D arrangement of the small air conduits and the air and blood capillaries
Mercox casting and SEM	3	Study of 3D arrangement of air conduits and gas exchange tissue

^*^The animals used for TEM, semithin, and SEM were the same ones used for enumeration of secondary bronchi. ^Ψ^Identification of the various categories was based on the sizes at their openings into the mesobronchus, positions of their openings, and also topographical disposition.

**Table 2:** Numbers of the various categories of secondary bronchi in the duck lung.

No.	MV		LD		LV		PO	
	L	R	L	R	L	R	L	R
1	4	4	7	10	2	2	44	40
2	4	5	7	8	2	2	39	40
3	4	4	10	10	4	4	42	38
4	5	5	6	8	3	4	40	37
5	4	4	8	8	4	2	38	36
6	5	5	8	8	4	2	44	42

Mean	4.3 ± 0.5	4.5 ± 0.5	7.7 ± 1.3	8.7 ± 0.9	3.2 ± 0.9	2.7 ± 0.9	41.2 ± 2	38.8 ± 2
Range	4-5	4-5	6–10	8–10	2–4	2–4	38–44	36–42

Explanations for abbreviations are as follows: LV: lateroventral; MV: medioventral; LD: laterodorsal; PO: posterior; R: right lung; L: left lung.

**Table 3:** Comparison of the numbers of secondary bronchi in the chicken lung with those of the duck lung. For the duck lung, averages for both the left and right lungs have been calculated from Table 2.

Category	Chicken∗		Duck	
	Range	Mean	Range	Mean
MV	NA	4	NA	4
LD	6–10	9.5 ± 1.6	6–10	8.2 ± 1.1
LV	1–3	1.4 ± 0.6	2–4	3 ± 0.9
PO	20–60	36.7 ± 12.9	38–44	38.8 ± 2

^*^Means and ranges calculated from both the left and right lungs from data in Makanya and Djonov [4].

## References

[1] Hazelhoff E. H. (1951). Structure and function of the lung of birds. *Poultry Science*.

[2] Bretz W. L., Schmidt-Nielsen K. (1971). Bird respiration: flow patterns in the duck lung. *Journal of Experimental Biology*.

[3] Farmer C. G., Sanders K. (2010). Unidirectional airflow in the lungs of alligators. *Science*.

[4] Makanya A. N., Djonov V. (2008). Development and spatial organization of the air conduits in the lung of the domestic fowl, gallus Gallus variant domesticus. *Microscopy Research and Technique*.

[5] Scheid P., Piiper J. (1972). Cross-current gas exchange in avian lungs: effects of reversed parabronchial air flow in ducks. *Respiration Physiology*.

[6] Scheid P. (1979). Mechanisms of gas exchange in bird lungs. *Reviews of Physiology Biochemistry and Pharmacology*.

[7] Scheid P., Slama H., Willmer H. (1974). Volume and ventilation of air sacs in ducks studied by inert gas wash out. *Respiration Physiology*.

[8] Maina J. N., Woodward J. D. (2009). Three-dimensional serial section computer reconstruction of the arrangement of the structural components of the parabronchus of the Ostrich, Struthio camelus lung. *Anatomical Record*.

[9] Woodward J. D., Maina J. N. (2008). Study of the structure of the air and blood capillaries of the gas exchange tissue of the avian lung by serial section three-dimensional reconstruction. *Journal of Microscopy*.

[10] Makanya A., Anagnostopoulou A., Djonov V. (2013). Development and remodeling of the vertebrate blood-gas barrier. *BioMed Research International*.

[11] Maina J. N. (2002). Structure, function and evolution of the gas exchangers: comparative perspectives. *Journal of Anatomy*.

[12] Fedde M. R. (1998). Relationship of structure and function of the avian respiratory system to disease susceptibility. *Poultry Science*.

[13] Scheid P. (1978). Estimation of effective parabronchial gas volume during intermittent ventilatory flow: theory and application in the duck. *Respiration Physiology*.

[14] Lasewski R. C., Farner D. S., King J. R., Parkes K. C. (1972). Respiration function in birds. *Avian Biology*.

[15] Makanya A. N., El-Darawish Y., Kavoi B. M., Djonov V. (2011). Spatial and functional relationships between air conduits and blood capillaries in the pulmonary gas exchange tissue of adult and developing chickens. *Microscopy Research and Technique*.

[16] Makanya A. N., Djonov V. G. (2009). Parabronchial angioarchitecture in developing and adult chickens. *Journal of Applied Physiology*.

[17] Nasu T. (2005). Scanning electron microscopic study on the microarchitecture of the vascular system in the pigeon lung. *Journal of Veterinary Medical Science*.

[18] Schachner E. R., Hutchinson J. R., Farmer C. (2013). Pulmonary anatomy in the Nile crocodile and the evolution of unidirectional airflow in Archosauria. *PeerJ*.

[19] Scheid P., Slama H., Piiper J. (1972). Mechanisms of unidirectional flow in parabronchi of avian lungs: measurements in duck lung preparations. *Respiration Physiology*.

[20] Akester A. R. (1960). The comparative anatomy of the respiratory pathways in the domestic fowl (*Gallus domesticus*), pigeon (*Columbia livia*) and domestic duck (*Anas platyrhyncha*). *Journal of anatomy*.

[21] Duncker H. R. (1971). The lung air sac system of birds. A contribution to the functional anatomy of the respiratory apparatus. *Ergebnisse der Anatomie und Entwicklungsgeschichte*.

[22] Lopez J., Gomez E., Sesma P. (1992). Anatomical study of the bronchial system and major blood vessels of the chicken lung (Gallus gallus) by means of a three-dimensional scale model. *Anatomical Record*.

[23] Kuethe D. O. (1988). Fluid mechanical valving of air flow in bird lungs. *Journal of Experimental Biology*.

[24] Bulacio R., Hazelhoff M. H., Torres A. M. (2012). Renal expression and function of oat1 and oat3 in rats with vascular calcification. *Pharmacology*.

[25] Eliason E. J. (2010). Alligators, like birds, breathe one way only. *The Journal of Experimental Biology*.

[26] Makanya A. N., Hlushchuk R., Djonov V. (2011). The pulmonary blood-gas barrier in the avian embryo: inauguration, development and refinement. *Respiratory Physiology & Neurobiology*.

[27] Powell F. L., Fedde M. R., Gratz R. K., Scheid P. (1978). Ventilatory response to CO2 in birds. I. Measurements in the unanesthetized duck. *Respiration Physiology*.

